# Clinical characterization and outcomes of 85 patients with neurosarcoidosis

**DOI:** 10.1038/s41598-021-92967-6

**Published:** 2021-07-02

**Authors:** Manuel Ramos-Casals, Roberto Pérez-Alvarez, Belchin Kostov, Ricardo Gómez-de-la-Torre, Carlos Feijoo-Massó, Joel Chara-Cervantes, Blanca Pinilla, Andrés González-García, José-Salvador Garcia-Morillo, Miguel López-Dupla, Begoña De-Escalante, Javier Rascón, Patricia Perez-Guerrero, Mariona Bonet, Gracia Cruz-Caparrós, Ana Alguacil, José-Luis Callejas, Eva Calvo, Cristina Soler, Angel Robles, Borja de Miguel-Campo, Pedro Oliva-Nacarino, Jorge Estela-Herrero, Lucio Pallarés, Pilar Brito-Zerón, Yolanda Blanco, the SarcoGEAS-SEMI Registry, M. Ramos-Casals, R. Pérez-Alvarez, B. Kostov, R. Gómez-de-la-Torre, C. Feijoo-Massó, J. Chara-Cervantes, B. Pinilla, A. González-García, J. S. Garcia-Morillo, M. López-Dupla, B. De-Escalante, J. Rascón, P. Perez-Guerrero, M. Bonet, G. Cruz-Caparrós, A. Alguacil, J. L. Callejas, E. Calvo, C. Soler, A. Robles, B. de Miguel-Campo, P. Oliva-Nacarino, J. Estela-Herrero, L. Pallarés, P. Brito-Zerón, Y. Blanco, M. Pérez-Conesa, E. Fonseca-Aizpuru, M. Akasbi, G. De-la-Red, E. Peral-Gutiérrez, J. F. Gómez-Cerezo, S. Rodríguez-Fernández, A. Gato, A. J. Chamorro, C. Morcillo, I. Ojeda, M. J. Vives, M. Penadés, M. De-Vicente, X. Bosch, M. Pérez-de-Lis, B. Gracia-Tello, Neera Toledo Samaniego, A. Sisó-Almirall

**Affiliations:** 1 Department of Autoimmune Diseases, ICMiD, Hospital Clínic, Barcelona, Spain; 2 Department of Medicine, University of Barcelona, Barcelona, Spain; 3 Department of Internal Medicine, Hospital Alvaro Cunqueiro, Vigo, Spain; 4 Primary Healthcare Transversal Research Group, IDIBAPS, Primary Care Center Les Corts, CAPSBE, Barcelona, Spain; 5 Department of Statistics and Operational Research, Universitat Politècnica de Catalunya, Barcelona, Spain; 6 Department of Internal Medicine, Hospital Universitario Central de Asturias (HUCA), Oviedo, Spain; 7 Department of Internal Medicine, Hospital Parc Tauli, Sabadell, Spain; 8 Department of Internal Medicine, Hospital Josep Trueta, Girona, Spain; 9 Department of Internal Medicine, Hospital Gregorio Marañón, Madrid, Spain; 10 Department of Internal Medicine, Hospital Ramon y Cajal, Madrid, Spain; 11 Department of Internal Medicine, Hospital Virgen del Rocio, Seville, Spain; 12 Department of Internal Medicine, Hospital Joan XXIII, Tarragona, Spain; 13 Department of Internal Medicine, Hospital Clínico, Zaragoza, Spain; 14 Department of Internal Medicine, Hospital Son Espases, Palma de Mallorca, Spain; 15 Department of Internal Medicine, Hospital Puerta del Mar, Cádiz, Spain; 16 Department of Internal Medicine, Althaia, Xarxa Assistencial de Manresa, Manresa, Spain; 17 Department of Internal Medicine, Hospital de Poniente de El Ejido, Almería, Spain; 18 Department of Internal Medicine, Hospital Virgen de la Salud, Toledo, Spain; 19 Department of Internal Medicine, Hospital San Cecilio, Granada, Spain; 20 Department of Internal Medicine, Hospital San Jorge, Huesca, Spain; 21 Department of Internal Medicine, Hospital Santa Caterina, Girona, Spain; 22 Department of Internal Medicine, Hospital La Paz, Madrid, Spain; 23 Department of Internal Medicine, Hospital 12 de Octubre, Madrid, Spain; 24 Department of Neurology. Hospital, Universitario Central de Asturias (HUCA), Oviedo, Spain; 25 Department of Neurology, Hospital Parc Tauli, Sabadell, Spain; 26 Systemic Autoimmune Diseases Unit, Department of Internal Medicine, Hospital CIMA-Sanitas, Barcelona, Spain; 27 Department of Neurology, Hospital Clínic, Barcelona, Spain; 28 Department of Internal Medicine, Hospital Miguel Servet, Zaragoza, Spain; 29 Department of Internal Medicine, Hospital de Cabueñes, Gijón, Spain; 30 Department of Internal Medicine, Hospital Infanta Leonor, Madrid, Spain; 31 Department of Internal Medicine, Hospital Esperit Sant, Santa Coloma de Gramenet, Spain; 32 Department of Internal Medicine, Hospital Virgen de la Macarena, Seville, Spain; 33 Department of Internal Medicine, Hospital Infanta Sofía, Madrid, Spain; 34 Department of Internal Medicine, Hospital de Barbanza, A Coruña, Spain; 35 Department of Internal Medicine, Complejo Hospitalario General, Albacete, Spain; 36 Department of Internal Medicine, Hospital Universitario de Salamanca, Salamanca, Spain; 37 Department of Internal Medicine, Hospital Valle del Guadiato, Córdoba, Spain; 38 Department of Internal Medicine, Parc Sanitari San Joan de Déu, Sant Boi, Spain; 39 Department of Internal Medicine, Hospital de Manises, Valencia, Spain; 40 Department of Internal Medicine, Hospital Nuestra Señora del Prado, Talavera de la Reina, Spain; 41 Department of Internal Medicine, ICMiD, Hospital Clinic, Barcelona, Spain; 42 Department of Anesthesiology, Complejo Hospitalario Universitario de A Coruña, A Coruña, Spain

**Keywords:** Immunology, Neuroimmunology

## Abstract

To analyze the frequency and clinical phenotype of neurosarcoidosis (NS) in one of the largest nationwide cohorts of patients with sarcoidosis reported from southern Europe. NS was evaluated according to the Diagnostic Criteria for Central Nervous System and Peripheral Nervous System Sarcoidosis recently proposed by Stern et al. Pathologic confirmation of granulomatous disease was used to subclassify NS into definite (confirmation in neurological tissue), probable (confirmation in extraneurological tissue) and possible (no histopathological confirmation of the disease). Of the 1532 patients included in the cohort, 85 (5.5%) fulfilled the Stern criteria for NS (49 women, mean age at diagnosis of NS of 47.6 years, 91% White). These patients developed 103 neurological conditions involving the brain (38%), cranial nerves (36%), the meninges (3%), the spinal cord (10%) and the peripheral nerves (14%); no patient had concomitant central and peripheral nerve involvements. In 59 (69%) patients, neurological involvement preceded or was present at the time of diagnosis of the disease. According to the classification proposed by Stern et al., 11 (13%) were classified as a definite NS, 61 (72%) as a probable NS and the remaining 13 (15%) as a possible NS. In comparison with the systemic phenotype of patients without NS, patients with CNS involvement presented a lower frequency of thoracic involvement (82% vs 93%, q = 0.018), a higher frequency of ocular (27% vs 10%, q < 0.001) and salivary gland (15% vs 4%, q = 0.002) WASOG involvements. In contrast, patients with PNS involvement showed a higher frequency of liver involvement (36% vs 12%, p = 0.02) in comparison with patients without NS. Neurosarcoidosis was identified in 5.5% of patients. CNS involvement prevails significantly over PNS involvement, and both conditions do not overlap in any patient. The systemic phenotype associated to each involvement was clearly differentiated, and can be helpful not only in the early identification of neurological involvement, but also in the systemic evaluation of patients diagnosed with neurosarcoidosis.

## Introduction

Sarcoidosis is an immune-mediated granulomatous systemic disease^1^ that affects adults with a slight predominance in women at a mean age around 40 years^2^, with an estimated prevalence ranging between 1 and 40 cases per 100,000 individuals^2,3^. Sarcoidosis may mimic a wide variety of processes, and the diagnosis is established when specific clinical and imaging findings are supported by histologic evidence of non-caseating granulomas^4^. Spontaneous remission may occur in up to two thirds of patients, while the remaining cases may follow a chronic course^5^.

The lungs and the thoracic lymph nodes are involved in nearly 90% of patients with sarcoidosis^6^. However, extrathoracic involvement is a key characteristic of the disease, and involvement of the nervous system (neurosarcoidosis) is one of the most characteristic systemic features involving around 3–9% of patients^7,8^. The clinical picture is heterogeneous since any part of the nervous system (brain, meninges, cranial nerves, spinal cord, peripheral nerves) can be affected^9^. Given the key role of histopathological examination for supporting the diagnosis of sarcoidosis, a major challenge regarding neurosarcoidosis is the difficulty of obtaining neural tissue, especially in those cases with involvement of the central nervous system. The diverse clinical presentation and the complex approach for obtaining neurological tissue make to consider neurosarcoidosis as one of the extrathoracic involvements more complex to diagnose and manage^10^.

Despite numerous publications on neurosarcoidosis, there is no international consensus about their definition and classification. A variety of diagnostic criteria have been proposed in the last 20 years with a wide methodological approach among them and therefore with significant limitations for being applied. A recent study from the Neurosarcoidosis Consortium Consensus Group has launched a consensus diagnostic set of criteria providing definitions for possible, probable and definite neurosarcoidosis^10^. In this study, we have analyzed the frequency and clinical phenotype of neurosarcoidosis in one of the largest nationwide cohorts of patients with sarcoidosis reported from southern Europe following these new diagnostic criteria.

## Methods

### Patients

The SARCOGEAS-Study Group was founded in 2015 with the aim of collecting a large series of patients with sarcoidosis from Spanish hospitals with substantial experience in the management of systemic autoimmune diseases^6^. Both incident and prevalent cases were included. By January 2020, the multicenter database included 1532 consecutive patients diagnosed with sarcoidosis in 40 centers according to the criteria proposed by the American Thoracic Society/European Respiratory Society/World Association of Sarcoidosis and Other Granulomatous Disorders (WASOG) 1999 statement on sarcoidosis^11^: (a) clinical or radiologic findings consistent with sarcoidosis, such as pulmonary disease, uveitis, mediastinal bilateral hilar lymphadenopathy (BHL), or erythema nodosum; (b) tissue biopsy with histologic evidence of non-caseating granulomas; (c) absence of other causes of granulomatous disease. Patients lacking the histopathological criteria (b) were included if they presented at least one of the following features: elevated serum angiotensin-converting enzyme, organ-specific abnormal uptake on gallium-67 citrate scintigraphy, elevated lymphocyte count or elevated CD4/CD8 ratio in bronchoalveolar lavage fluid, or active extrathoracic involvement classified as highly probable according to the WASOG extrathoracic classification^12,13^. Extrathoracic involvement at diagnosis was defined according to the 2014 WASOG organ assessment instrument, including only the clinical scenarios classified as highly probable or at least probable^13^. The study was conducted in accordance with the amended Declaration of Helsinki. The Ethics Committee of the coordinating center (Clinical Research Ethics Committee of the Hospital Clinic, Barcelona, Spain, code HCB2016/0181) approved the protocol, and written informed consent was obtained from patients with current follow-up.

### Variables

Patients included in the Registry as having neurosarcoidosis according to the 2014 WASOG definition were selected. These cases were re-evaluated according to the Proposed Diagnostic Criteria for Central Nervous System and Peripheral Nervous System Sarcoidosis recently reported by Stern et al.^10^. The mandatory inclusion criteria consisted of a clinical presentation and diagnostic evaluation suggesting neurosarcoidosis, as defined by the clinical manifestations and magnetic resonance imaging (MRI), cerebrospinal fluid (CSF), and/or electromyogram/nerve conduction study (EMG/NCS) findings suggestive of granulomatous inflammation of the nervous system. Exclusion criteria included a rigorous exclusion of other causes of neurological disease. Pathologic confirmation of granulomatous disease was used to subclassify neurosarcoidosis into definite (non-caseating granulomas demonstrated in neurological tissue), probable (non-caseating granulomas demonstrated in extraneurological tissue) and possible (no histopathological confirmation of the disease)^10^ (Sup. Figure 1).

The following variables were retrospectively collected at the time of diagnosis of neurosarcoidosis, defined as the date of fulfilment of the Stern criteria: age, sex, signs and symptoms at neurological presentation, ACE serum levels, Scadding radiographic stages^14^, neurological diagnostic tests (MRI, CSF, EMG/NCS, PET), histopathological study, treatment and outcomes. The neurological diagnosis was achieved in each center after a multidisciplinary evaluation including neurologists, internists and radiologists, and was independently re-evaluated by MRC and YB, who applied the classification criteria and the subclassification into the 5 anatomical clusters proposed by Stern et al.^10^ (Sup. Table 1), requiring to the participant centers additional information in case was necessary to confirm the fulfilment of the Stern criteria.

### Statistical analysis

Descriptive data are presented as mean and standard deviation (SD) for continuous variables and numbers and percentages (%) for categorical variables. Phenotyping comparisons were made considering the presence or absence of neurosarcoidosis, and among the two main subsets of neurosarcoidosis (peripheral and central nervous system involvement). The Chi-square test was used to study the association between Scadding subsets with the main epidemiological, clinical, extrathoracic and therapeutic variables. One-way ANOVA tests were used to compare continuous variables. All significance tests were two-tailed and values of p < 0.05 were considered significant. P-values were adjusted for multiple comparisons using the false discovery rate (FDR) correction. All analyses were conducted using the R V.3.2.3 for Windows statistical software package (R: A Language and Environment for Statistical, R Core Team, R Foundation for Statistical Computing, Vienna, Austria, 2015, https://www.R-project.org/).

## Results

Of the 1532 patients included in the cohort, 102 were reported as having neurosarcoidosis according to the WASOG criteria. Among them, 17 patients were excluded after applying the Stern criteria due to lack of enough information for applying the criteria (n = 11), lack of abnormal neurological diagnostic tests (n = 4) or other etiologies unrelated to sarcoidosis (n = 2). Therefore, the remaining 85 (5.5%) of patients fulfilled the Stern criteria for neurosarcoidosis and were specifically analysed. There were 49 (58%) women and 36 (42%) men, with a mean age at diagnosis of neurosarcoidosis of 47.6 years (range 22–76); all patients except 8 were classified as White. Scadding radiologic stage consisted of stage 0 in 15 (18%) patients, stage I in 28 (33%), stage II in 31 (36%), stage III in 9 (11%) and stage IV in 2 (2%) patients. Histopathological demonstration of non-caseating granulomas was reported in 72 (85%) patients. In addition to the neurological involvement, 59 (69%) patients presented other extrathoracic manifestations and 21 (25%) presented only with thoracic manifestations; in the remaining 5 (6%) patients, the nervous system was the unique organ involved by sarcoidosis at the time of diagnosis (Table 1).

**Table 1:** Epidemiological profile, clinical features and outcomes of 85 patients with neurosarcoidosis defined according to the Stern criteria.

	N = 85	Frequency (%)
Women	50	59
Mean age NS	47.6	
White patients	78	92
**Scadding radiological stages**		
Stage 0	15	18
Stage I	28	33
Stage II	31	26
Stage III	9	11
Stage IV	2	2
Raised ECA	41/74	55
Bx-proven diagnosis	72	85
NRL presentation	59	69
**Systemic phenotypes**		
Isolated thoracic disease	21	25
Extrathoracic, non-NRL	59	69
Isolated NS	5	6
**Neurosarcoidosis classification**		
Definitive	11	13
Probable	61	72
Possible	13	15
**Neurosarcoidosis involvement**^a^		
Brain	39	38
Cranial nerves	37	36
Spinal cord	10	10
Meningitis	3	3
Peripheral nerves	14	14
Multiple neurological anatomical sites involved	16	19
**Treatment**		
Corticosteroids	76/83	92
Immunosuppresant agents	27/83	33
Biologics	3/83	4
**Therapeutic response**		
Complete response	47/78	60
Partial response	22/78	28
No response	9/78	12
**Outcomes**		
Relapse of NS	7/68	10
Death caused by NS	0	0

^a^Patients may have more than one involvement.

### Anatomical involvements

The 85 patients with neurosarcoidosis developed 103 neurological conditions involving the brain (n = 39, 38%), cranial nerves (n = 37, 36%), the meninges (n = 3, 3%), the spinal cord (n = 10, 10%) and the peripheral nerves (n = 14, 14%). In 59 (69%) patients, neurological involvement preceded or was present at the time of diagnosis of the disease. Raised serum ECA levels were reported in 41/74 (55%) of cases. Forty-two patients had cerebrospinal fluid (CSF) analysis. Eight had completely normal CSF constituents. The most consistent abnormality was an elevated CSF protein (33/42 patients, 79%), followed by CSF pleocytosis (21/42 patients, 50%, with a range of 11–110 white cells, overwhelmingly monocytes). There were two cases of mild hypoglycorrhachia, in whom extensive microbiological studies were negative. Oligoclonal bands were studied in 8 patients (negative in 5, matched positive in 2 and positive with intrathecal synthesis alone in one). The CSF ACE level was measured in only one patient (normal levels).

Table 2 summarizes the demographics and therapeutic information of patients with neurosarcoidosis according to the different anatomical phenotypes.

**Table 2:** Demographics and therapeutic information of patients with neurosarcoidosis according to the different anatomical phenotypes.

	Brain involvement (n = 39)	Cranial nerve involvement (n = 37)	Meningeal involvement (n = 3)	Spinal cord involvement (n = 10)	Neuropathy (n = 14)
Sex (women)	25 (64%)	25 (68%)	1 (33%)	5 (50%)	5 (36%)
Mean age at diagnosis (mean)	46.1 years	47.1 years	50.0 years	45.3 years	50.4 years
**Neurological presentation**					
Preceded/coincident with sarcoidosis diagnosis	27 (69%)	26 (70%)	1 (33%)	10 (100%)	11 (79%)
Nervous system as the only organ involved	4 (10%)	1 (3%)	0 (0%)	1 (10%)	0 (0%)
**Neurosarcoidosis classification (Stern et al.)**					
Definitive	8 (21%)	2 (5%)	0 (0%)	0 (0%)	2 (14%)
Probable	25 (64%)	27 (73%)	3 (100%)	8 (80%)	11 (79%))
Possible	6 (15%)	8 (22%)	0 (0%)	2 (20%)	1 (7%)
Multiple neurological anatomical sites involved	15 (38%)	12 (32%)	2 (67%)	5 (50%)	0 (0%)
**Treatment**					
Corticosteroids	37 (95%)	34 (92%)	3 (100%)	10 (100%)	10 (71%)
Immunosuppresant/biologic agents	14 (36%)	11 (30%)	1 (33%)	5 (50%)	2 (14%)
**Therapeutic response**					
Complete response	22/36 (61%)	23/35 (66%)	2 (67%)	3 (30%)	5/11 (45%)
Partial response/no response	14/36 (39%)	12/35 (34%)	1 (33%)	7 (70%)	6/11 (55%)
Relapse in complete responders	6/22 (27%)	1/23 (4%)	0 (0%)	1/3 (33%)	0/5 (0%)

According to the classification proposed by Stern et al., 11 (13%) were classified as a definite neurosarcoidosis, 61 (72%) as a probable neurosarcoidosis and the remaining 13 (15%) as a possible neurosarcoidosis (Table 1).

#### Brain involvement

**Figure 1:**
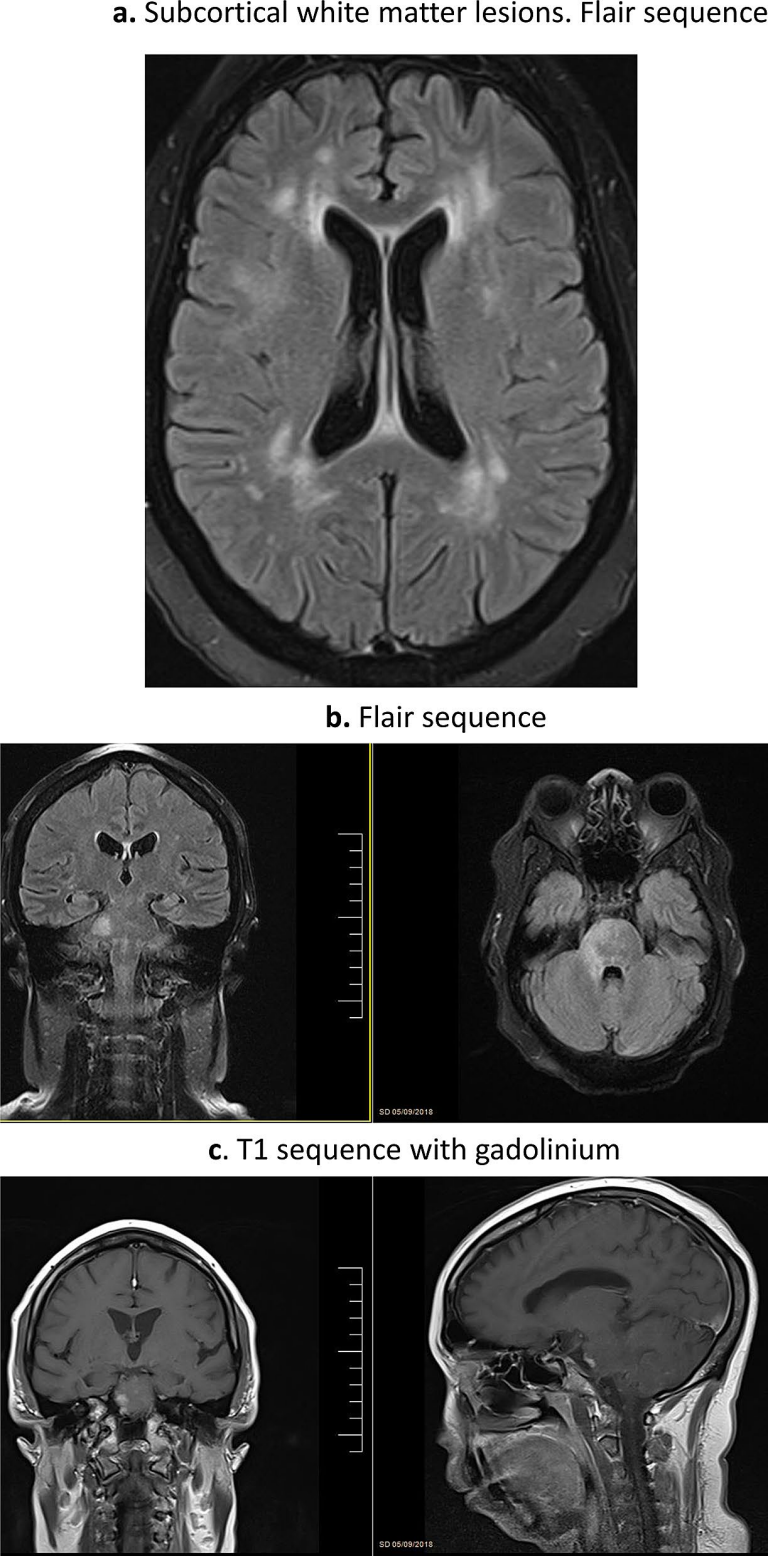
Intraparenchymal lesions. Pontine right lateral lesion that enhances with gadolinium: (**a**) Flair sequence and (**b**) T1 sequence with gadolinium; (**c**) Subcortical white matter lesions, Flair sequence.

**Figure 2:**
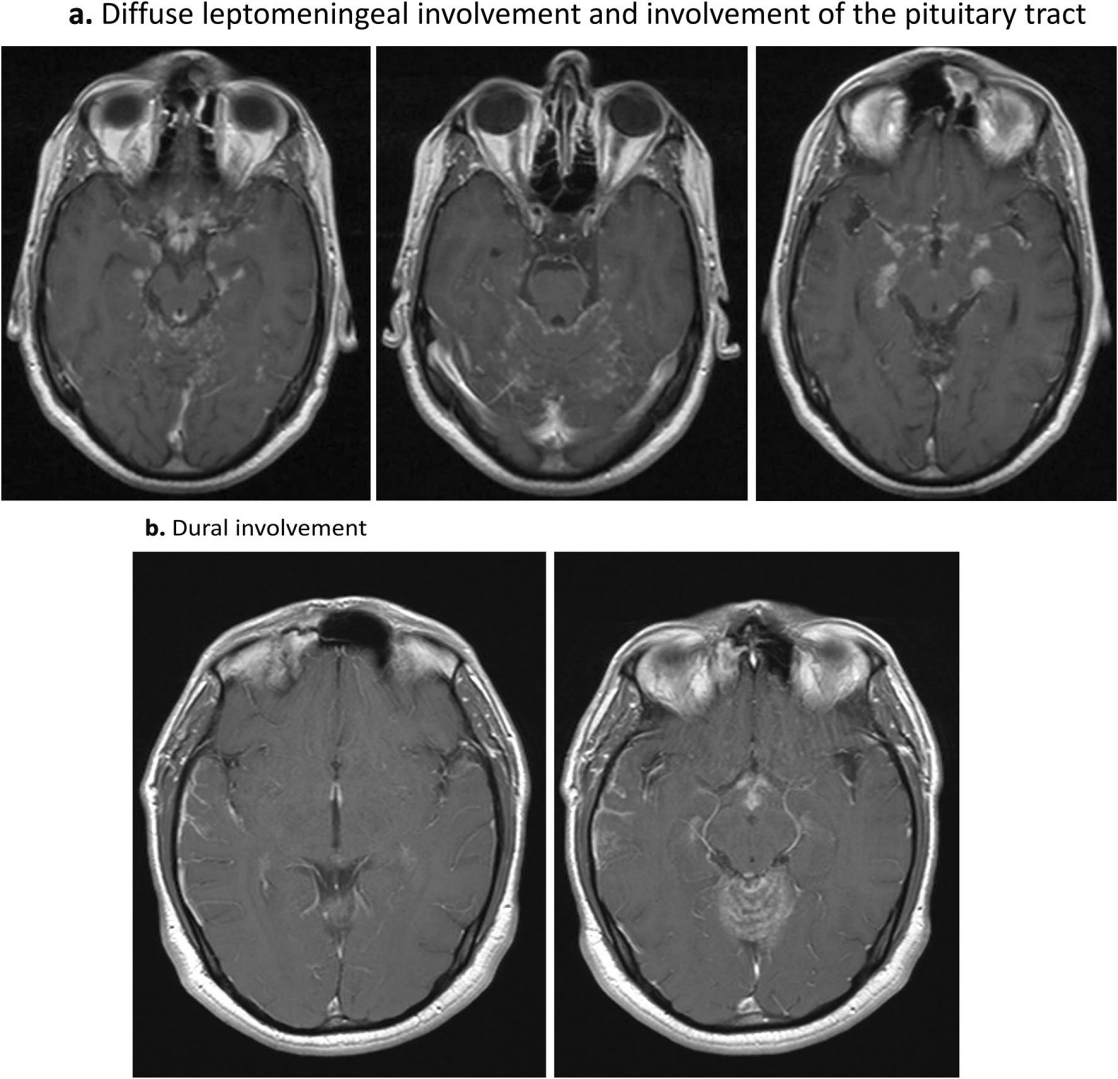
Leptomeningeal and dural involvement. (**a**) Diffuse leptomeningeal involvement and involvement of the pituitary tract; (**b**) Dural involvement.

Brain involvement was reported in 39 patients (25 women, mean age at diagnosis of neurosarcoidosis of 46.1 years). Neurological involvement preceded or was present at the time of diagnosis of sarcoidosis in 27 (69%) cases, and the nervous system was the only organ involved in 4 (10%). Clinical neurological presentation consisted of focal motor deficits (n = 14), headache (n = 12), clinical involvement of cranial nerves (n = 8), confusion/cognitive impairment (n = 7), seizures (n = 6), polyuria/polydipsia (n = 3), signs and symptoms of ICH (n = 2) and fever (n = 1); the neurological signs and symptoms often overlapped. Neurological imaging studies disclosed intraparenchymal brain lesions in 23 cases consisting of small subcortical or periventricular white matter lesions (n = 20) (Fig. 1a) or 1–2 large isolated lesions (n = 3) (Fig. 1b,c), leptomeningeal involvement in 18 (especially along the skull base, including the involvement of the pituitary tract in 6 patients) (Fig. 2a), dural involvement in 5 (Fig. 2b) and hydrocephalus in 2 cases; no patient showed stroke or intracerebral haemorrhage. Other diagnostic tests included abnormal PET uptake of the nervous system in 2/11 (18%) and abnormal CSF results in 22/25 (88%). There were 15 patients that presented with other concomitant anatomical neurological involvements (cranial nerves in 11, myelitis in 5, meningitis in one). According to the classification proposed by Stern et al., 8 (21%) were classified as a definite neurosarcoidosis, 25 (64%) as a probable neurosarcoidosis and the remaining 6 (15%) as a possible neurosarcoidosis. Frontline therapeutic approach included the use of corticosteroids in all patients except 2, including intravenous administration in 10 (methylprednisolone pulses in 8, dexamethasone in 2) and oral administration in the remaining 25 (with a dose higher than 30 mg/day in 22 cases). Immunosuppressants were used in 13 patients (methotrexate in 7, azathioprine in 3, mycophenolate in 2, cyclophosphamide in 2) and biological therapies in 5 (infliximab in 4, adalimumab in 1, rituximab in 1); infliximab was stopped in 2 cases due to multiple episodes of infections (n = 1) and tuberculosis (n = 1). Data from therapeutic response was available in 36 cases showing a complete response in 21 (58%) cases, partial response in 13 (36%) and no response in 2 (6%). A relapse was reported in 6 patients, including 2 asymptomatic patients in neuroradiological studies disclosed disease progression.

#### Cranial nerve involvement

**Figure 3:**
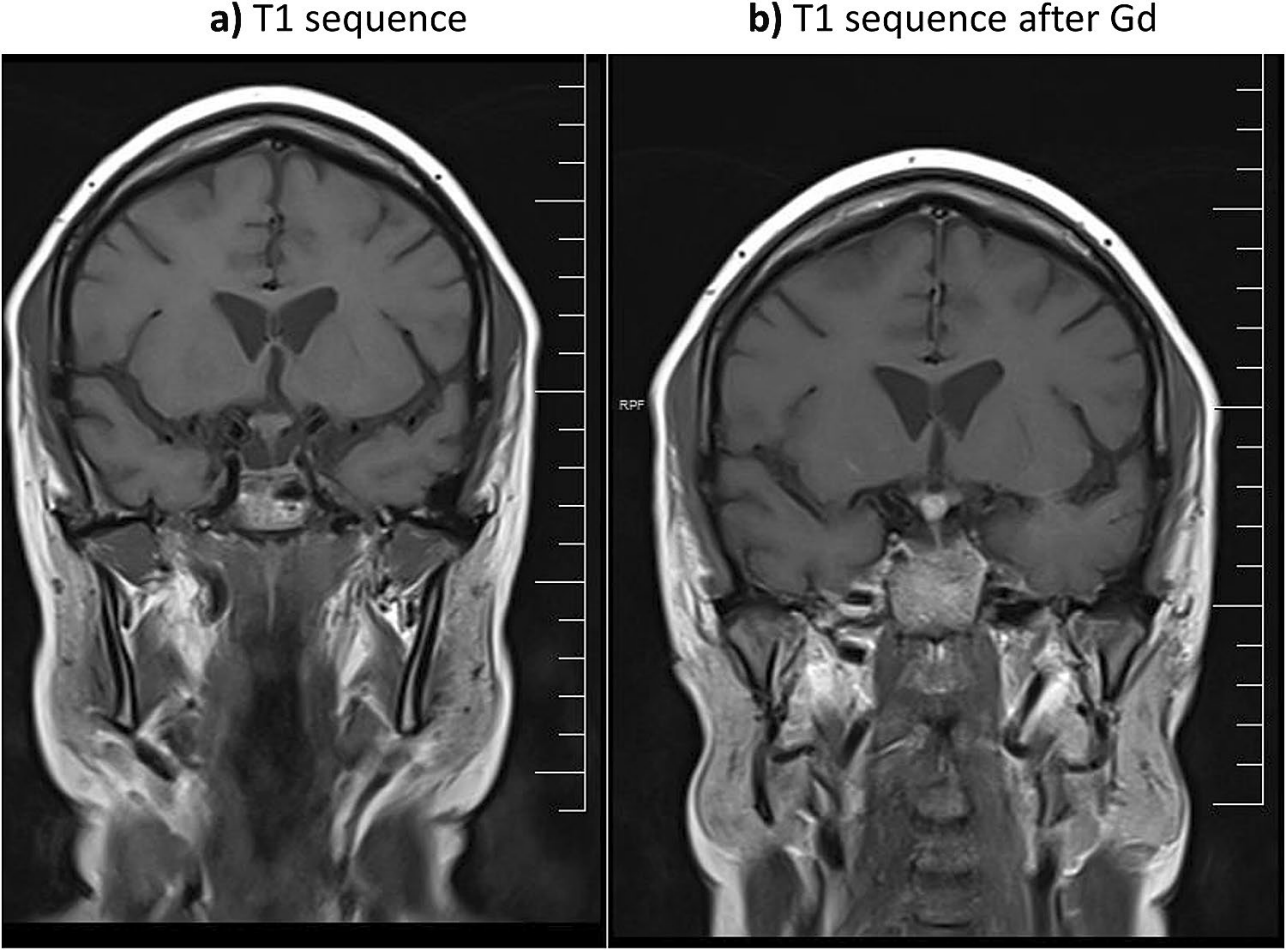
Involvement of optic chiasm and hypophysis; (**a**) T1 sequence; (**b**) T1 sequence after gadolinium.

Cranial nerve involvement was reported in 37 patients (25 women, mean age at diagnosis of neurosarcoidosis of 47.1 years). Neurological involvement preceded or was present at the time of diagnosis of sarcoidosis in 26 (70%) cases, and the nervous system was the only organ involved in only 1 (3%) case. Clinical neurological presentation consisted of signs and symptoms suggestive of involvement of the VII (n = 24), II (n = 5), V (n = 2) and VI (n = 1) cranial nerves; the remaining 5 cases presented with multiple cranial nerve involvement (3 cases involving the ocular cranial nerves, one case involving the VII and XII and one involving the IX and X). Neurological imaging studies disclosed involvement of the optic nerves (Fig. 3), together with other neurological involvements in 12 cases, mainly brain involvement in 11. Other diagnostic tests included abnormal PET uptake of the nervous system in 1/8 (12%) cases and abnormal CSF results in 10/15 (67%).

According to the classification proposed by Stern et al., 2 (5%) were classified as a definite neurosarcoidosis, 27 (73%) as a probable neurosarcoidosis and the remaining 8 (22%) as a possible neurosarcoidosis. Frontline therapeutic approach included the use of corticosteroids in all patients except 3, including methylprednisolone pulses in 5, and oral administration in 29 (with a dose higher than 30 mg/day in all cases). Immunosuppressants were used in 11 patients (methotrexate in 6, azathioprine in 2, mycophenolate in 2, cyclophosphamide in 2) and biological therapies in 2 (infliximab and rituximab, respectively). Data from therapeutic response was available in 35 cases in included a complete response in 23 (66%), partial improvement in 9 (26%) and no response in 3 (8%). Only 1 patient relapsed during the follow-up.

#### Meningeal involvement

Three patients presented with lymphocytic meningitis (1 woman, mean age at diagnosis of neurosarcoidosis of 50 years); in one case, neurological involvement preceded or was present at the time of diagnosis confirmation of the disease. All patients presented signs and symptoms of meningism (one together with paraparesia) and abnormal CSF studies, with normal neuroradiological studies. There were 2 patients that showed other concomitant anatomical neurological involvements. According to the classification proposed by Stern et al., all cases were classified as probable neurosarcoidosis. Frontline therapeutic approach included the use of corticosteroids with a dose higher than 30 mg/day in all patients, and azathioprine was added in one case. A complete response was reported in 2 cases, while the remaining patient showed a partial response. One patient developed a haemorrhagic stroke during the follow-up.

#### Spinal cord involvement

**Figure 4:**
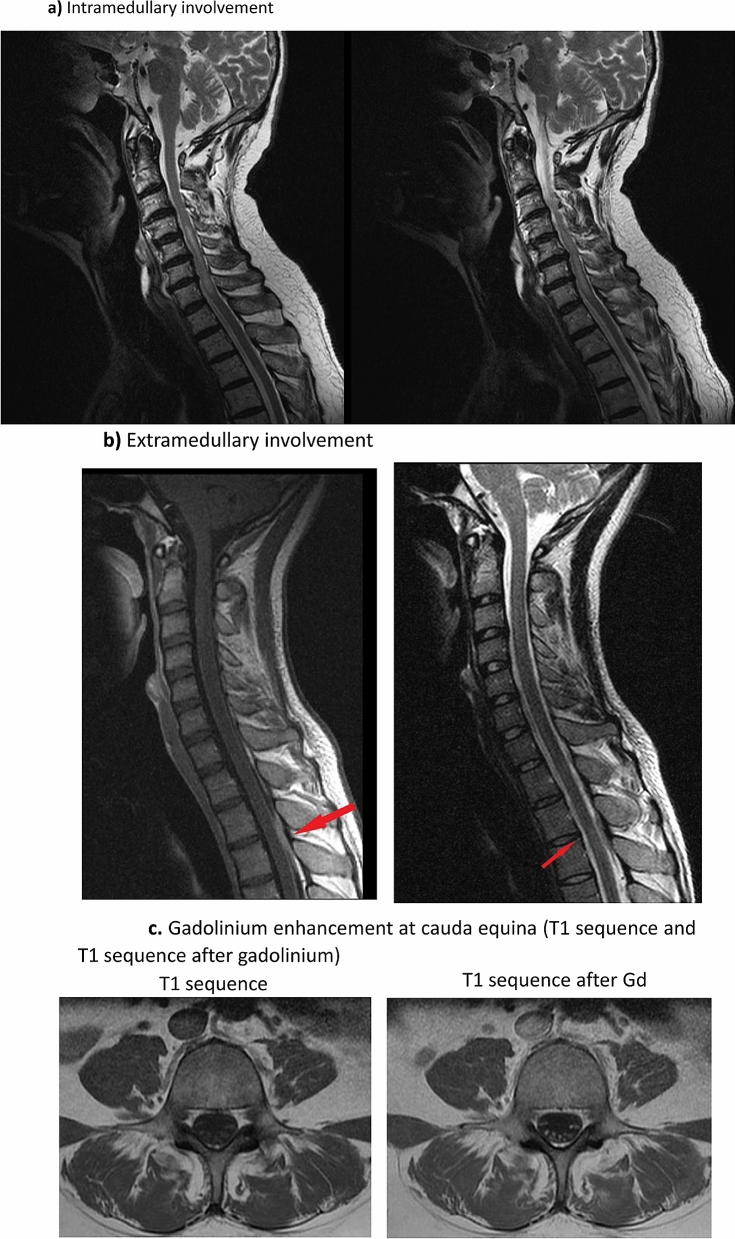
Medullary involvement: (**a**) Intramedullary involvement; (**b**) Extramedullary involvement; (**c**) Gadolinium enhancement at cauda equina (T1 sequence and T1 sequence after gadolinium).

Spinal cord involvement was reported in 10 patients (5 women, mean age at diagnosis of neurosarcoidosis of 45.3 years). In all cases, neurological involvement preceded or was present at the time of diagnosis confirmation of the disease, and the nervous system was the only organ involved in only 1 (10%) case. Clinical neurological presentation consisted of focal motor deficits (n = 9), headache (n = 3), meningism (n = 1) and cranial nerve involvement (n = 1). Neurological imaging studies disclosed intramedullary **(**Fig. 4a) or extramedullary involvement (Fig. 4b) (with dural enhancement on 4 cases) located in the cervical region (n = 3), the cervical and dorsal regions (n = 2), the dorsal region (n = 2) and the conus medullaris/cauda equina region (n = 2) (Fig. 4c); enhancement extended for more than 3 segments in 5 patients. Abnormal CSF results were reported in 7/8 (88%). There were 5 patients that showed concomitant brain lesions. According to the classification proposed by Stern et al., 8 (80%) were classified as a probable neurosarcoidosis and the remaining 2 (20%) as a possible neurosarcoidosis. Frontline therapeutic approach included the use of corticosteroids with a dose higher than 30 mg/day in all patients (including methylprednisolone pulses in one case). Immunosuppressants were used in 5 patients (methotrexate in 3, mycophenolate in 2, cyclophosphamide in 1) and biological therapies in 1 (infliximab). Data from therapeutic response showed a complete response in 3 (30%), partial improvement in 6 (60%) and no response in 1 (10%, no response to MMF, IFX started but stopped for infections). One patient relapsed at 6-months of follow-up during corticosteroid tapering.

#### Neuropathy

Peripheral nerve involvement was reported in 14 patients (5 women, mean age at diagnosis of neurosarcoidosis of 50.4 years). In 11 cases, neurological involvement preceded or was present at the time of diagnosis confirmation of the disease. Clinical neurological presentation consisted of signs and symptoms suggestive of sensory neuropathy in 12 cases and radicular paresthesia/paraparesis in the remaining 2. Electromyogram/nerve conduction studies disclosed axonal polyneuropathy (n = 7), pure sensitive neuropathy/small fiber neuropathy (n = 4), polyradiculopathy (n = 2) and inconclusive, non-classified polyneuropathy (n = 1). Neurological imaging studies were normal in 7/7. Other diagnostic tests included abnormal CSF results in 6/7 (86%). No patients showed concomitant involvement of the central nervous system. According to the classification proposed by Stern et al., 2 (14%) were classified as a definite neurosarcoidosis, 11 (79%) as a probable neurosarcoidosis and the remaining one (7%) as a possible neurosarcoidosis. Frontline therapeutic approach included the use of corticosteroids in 10 (71%) cases, including methylprednisolone pulses in 2, and oral administration in 8 (with a dose higher than 30 mg/day in 6 cases). Immunosuppressants were used in 2 patients (methotrexate) and IVIG in 1. Data from therapeutic response was available in 11 cases and included a complete response in 5 (45.5%), partial improvement in 1 (9%) and no response in 5 (45.5%).

### Comparison studies

#### Differences according to anatomical involvement

No significant differences were found between patients with CNS involvement and those with PNS involvement except for a trend for a higher frequency of women in patients with CNS involvement and a higher frequency of renal involvement in those with PNS involvement (14% vs 0%, p = 0.025) (Table 3). In patients with CNS involvement, no significant differences were found between those with brain involvement and those with CNS involvement out of the brain except for a higher frequency of multiple anatomical involvements in patients with brain involvement (38% vs 3%, p < 0.001) (Sup. Table 2). Patients presenting with a unique CNS neurological involvement showed a higher frequency of raised ECA levels (62% vs 29%, p = 0.036) and a lower frequency of salivary gland involvement (10% vs 31%, p = 0.044) in comparison with those with multiple CNS sites involved (Sup. Table 3).

**Table 3:** Phenotypic differences according to the presence or absence of neurosarcoidosis in patients with CNS neurosarcoidosis and in those with PNS neurosarcoidosis.

	CNS involvement (n = 71)	Neuropathy (n = 14)	No neurosarcoidosis (n = 1430)
Sex (women)	44 (62)	5 (35.7)	828 (57.9)
Mean age sarco	45.5 ± 13.9	46.9 ± 18.8	47.5 ± 15.2
Etnia (W vs no-W)	64 (90.1)	13 (92.9)	1286 (89.9)
**Scadding radiological stages**			
Stage 0	13 (18.3)^^^*	2 (14.3)	104/1412 (7.4)
Stage I/II	48 (67.6)	11 (78.6)	986/1412 (69.8)
Stage III/IV	10 (14.1)	1 (7.1)	322/1412 (22.8)
Raised ECA	31/60 (51.7)	10 (71.4)	625/1121 (55.8)
Bx-proven dx	59 (83.1)	13 (92.9)	1164 (81.4)
**WASOG extrathoracic involvement**			
Cutaneous (≥ 1)	19 (26.8)	5 (35.7)	533 (37.3)
Lymph nodes (≥ 1)	14 (19.7)	4 (28.6)	288 (20.1)
Ocular (≥ 1)	19 (26.8)^^^*	2 (14.3)	137 (9.6)
Liver (≥ 1)	11 (15.5)	5 (35.7)^^^	170 (11.9)
Spleen (≥ 1)	5 (7)	3 (21.4)	107 (7.5)
Salivary (≥ 1)	11 (15.5)^^^*	1 (7.1)	63 (4.4)
ENT (≥ 1)	3 (4.2)	0 (0)	32 (2.2)
Articular/bone (≥ 1)	3 (4.2)	0 (0)	102 (7.1)
Muscular (≥ 1)	0 (0)	1 (7.1)	18 (1.3)
Renal (≥ 1)	0 (0)	2 (14.3)^º^	55 (3.8)
Ca/Vit D (≥ 1)	5 (7)	2 (14.3)	127 (8.9)
Heart (≥ 1)	0 (0)	0 (0)	29 (2)
**Treatment**			
Corticosteroids	66/69 (95.7)^^^*	10 (71.4)^º^	672 (47)
Immunosuppresant/biologic agents	24/69 (34.8)^^^*	2 (14.3)	115 (8)

^^^p value < 0.05 in comparison with patients without neurosarcoidosis.

*q value < 0.05 in comparison with patients without neurosarcoidosis.

^º^p value < 0.05 in comparison with patients with CNS involvement.

#### Comparison with patients without neurosarcoidosis

Patients with CNS neurosarcoidosis presented a higher frequency of radiological Scadding Stage 0 (18% vs 7%, q = 0.018), a higher frequency of ocular (27% vs 10%, q < 0.001) and salivary gland (15% vs 4%, q = 0.002) WASOG involvements, and a higher frequency of use of corticosteroids (96% vs 47%, q < 0.001) and immunosuppressive/biological agents (35% vs 8%, q < 0.001) in comparison with patients without neurosarcoidosis, while those with PNS neurosarcoidosis showed a higher frequency of liver involvement (36% vs 12%, p = 0.02) and a trend for a higher frequency of spleen involvement (21% vs 7%, p = 0.08) that were not statistically significant in the corrected statistical analysis (Table 3).

## Discussion

Neurosarcoidosis is a potentially life-threatening presentation of sarcoidosis that has been reported in 3–9% of patients with sarcoidosis^7,8,15–18^. There are few large studies focusing on the characterization of this extrathoracic presentation of sarcoidosis, with only 7 series including more than 30 cases that have been published in the last 20 years, mainly from the US^19–23^ and Northern Europe^9,24^ (Sup. Table 4). In this study, we have characterized the largest series of patients with neurosarcoidosis from Southern Europe, and the second one reported worldwide, using for the first time the new diagnostic criteria proposed by Stern et al.^10^. Neurosarcoidosis was identified in 5.5% of our patients with sarcoidosis, a similar figure than that reported in previous cohorts of patients^7,8,15–18^.

According to previous studies (Sup. Table 4), patients with neurosarcoidosis are women in 44–77% of cases, with a frequency of non-White people ranging between 40 and 96%, and a mean age at diagnosis of the neurological disease ranging from 40 to 48 years; neurosarcoidosis is present at the time of diagnosis of sarcoidosis in 60–72% of cases, and is the single organ involved in 3–20% of reported cases. The epidemiological profile in our series was quite similar (59% women, mean age at diagnosis around 48 years), and also the timing of presentation (neurosarcoidosis was present at the time of diagnosis in 59% of cases being the single organ involved in 6%). We report one of the oldest mean age at diagnosis of neurosarcoidosis among the large reported series, a fact that could be linked with having the reported series with the highest frequency of White people (92% of our cases, in comparison with only 4–60% of patients included in previous studies) (Sup. Table 4), since sarcoidosis is diagnosed at older ages in White patients in comparison with other ethnicities^2^.

Among patients with CNS involvement, we found a clear predominance of brain (55%) and cranial nerve (52%) involvements, and a low frequency of spinal cord (14%) and meningeal (4%) involvements. The main studies show that brain (33–52%) and cranial nerve (31–76%) involvements also are the predominant presentation of neurosarcoidosis^18,19,22,24–30^, as has also been confirmed in a recent systematic review analysing 652 patients with neurosarcoidosis (parenchymal brain lesions were reported in 51% of cases and cranial neuropathies in 55%)^31^. We found that intraparenchymal lesions and leptomeningeal involvement were the key features of sarcoidosis involving the brain, as has reported by previous studies^9,22^; we also found involvement of the pituitary tract, a very specific site involved by neurosarcoidosis^32^, in 9% of patients, a frequency that is into the range of that previously reported (2–25%)^19,21–24^. Pathological studies in patients with sarcoidosis involving the brain showed microvascular changes (involving small arteries, arterioles and small veins) and granulomatous inflammation often following a perivascular distribution^26^, as we could see in 3 of our patients in whom brain biopsy was carried out. Brain neurosarcoidosis is characterized by a mixed cellular infiltration composed by histiocytes, macrophages, both CD4 and CD8 T lymphocytes and B lymphocytes. In our patients with brain involvement, we reported a complete/partial response in more than 90% of cases; in previous studies^19,21–24^, we cannot found specific data about the therapeutic response in patients with sarcoidosis involving the brain, since the main series do not separate the therapy and outcome of these patients from other patients with neurosarcoidosis.

We found cranial nerve involvement in half of our patients with neurosarcoidosis; involvement of the facial nerve represented 65% of all cranial neuropathies we identified. In the systematic review by Fritz et al.^31^, the facial nerve was also the most frequent cranial nerve involved (40%), followed by the optic, trigeminal and audiovestibular nerves. In some cases, the facial palsy is included into a Heerfordt’s syndrome presentation together with uveitis, parotid gland enlargement and fever. Sarcoidosis may also cause an optic neuritis, which is vision-threatening, and has a poorer GC response and outcome in comparison with other cranial nerves involved. In the 5 cases of optic neuritis that we reported, the rate of response to corticosteroids monotherapy was 40%, and most patients required the addition of immunosuppressive/biologic agents to improve the response, and a complete response was achieved in only one case. Previous studies have reported a therapeutic response to corticosteroids in 15 (52%) out of 29 patients with sarcoidosis and optic neuritis^22,33,34^, with a worse prognosis in those presenting with a chronic course or with bilateral involvement.

Spinal cord sarcoidosis is a rare but potentially life-threatening involvement that may present with paraplegia, paralysis, incontinence, and cauda equina syndrome, mimicking NMOSD^35^. Neuroimaging studies may disclose a wide spectrum of findings, including arachnoiditis, extradural or intradural lesions, and intramedullary involvement^36^. Among the 10 cases we have reported, most showed intramedullary involvement, as has been reported in previous studies^25,36^; these patients were treated with high doses of corticosteroids achieving a complete response in only 30% of cases. In previous studies, the frequency of complete response is also low, and more than 80% of cases had neurological sequelae at the end of the follow-up^25,36–38^. Meningeal involvement of sarcoidosis was the less frequent CNS involvement in our series, with only 3 cases.

PNS involvement was identified in 14% of our patients diagnosed with neurosarcoidosis according to Stern criteria^10^. Involvement of peripheral nerves by sarcoidosis has been little studied in comparison with CNS involvement, with some small series (10 cases or less) being reported in the last 30 years^39–42^. The largest study has been recently reported by Tavee et al.^43^, who reported small fiber neuropathy in 115 (7%) patients out of 1580 patients assessed in the sarcoidosis clinic at the Cleveland Clinic (including patients with a clinical suspicion and those with abnormal tests/skin biopsy). In contrast, the frequency of PNS involvement in our cohort was lower than 1%, probably due to a lack of systematic study searching for NNS in patients with normal electromyographic studies; in addition, the criteria proposed by Stern et al.^10^ do not include specifically NNS in the definition of peripheral nerve involvement of sarcoidosis.

One of the objectives of our study was to analyse how different could be the disease phenotype of the different clinical subsets of neurosarcoidosis. First, we have analysed potential differences between the two main subtypes of neurosarcoidosis (CNS and PNS involvements) and we found that, interestingly, the two subtypes do not overlap in any patient, as has been reported in other systemic autoimmune diseases such as lupus^44^ or Sjögren syndrome^45^. However, we found no significant differences in the disease phenotype between patients with CNS involvement and PNS involvement, except for a statistically-significant trend for a higher frequency of women in patients with CNS involvement and a higher frequency of renal involvement in those with PNS involvement (an association rarely reported until now)^46^. In contrast, the phenotypic differences were more pronounced when these two neurological subsets were compared with patients without neurosarcoidosis. Patients with CNS sarcoidosis presented with a specific phenotype defined by a lower frequency of thoracic involvement and a higher frequency of other concomitant systemic involvements (ocular and salivary gland involvement), while those with PNS involvement showed a higher frequency of liver/spleen involvement in comparison with patients without neurosarcoidosis. These results confirm that systemic involvement in sarcoidosis usually clusters the involvement of some specific organs^47^, with five distinct clinical phenotypes recently described, including a cluster called ocular–cardiac–cutaneous–CNS (“OCCC”; eye/heart/skin/salivary glands/CNS), in which a statistical association was found with the concomitant development of neurological, ocular and salivary gland involvements.

Since neurosarcoidosis may be associated with a significant neurological impairment, it sounds reasonable to recommend treating all patients^48,49^. However, a systematic review^31^ report no specific therapeutic intervention in 15% of patients, probably in those with milder involvements. In our series, a wait and see management was followed in 7% of patients (in patients with isolated facial palsy or mild peripheral neuropathy), of whom only one showed a spontaneous partial recovery during the follow-up. Corticosteroids are well stablished as the first line therapy of neurosarcoidosis, and were administered in 83% of cases included in the systematic review by Fritz et al.^31^, followed by oral immunosuppressive agents as second line therapies in 27% and TNF-alpha antagonists in 4%. In our series, the frequencies were similar for the use of biologics (4%) and somewhat higher for the use of corticosteroids (92%) and immunosuppressive agents (32%). CNS sarcoidosis is one of the extrathoracic involvements with a worse prognosis, and the ultimate target is to achieve a clinical improvement, or at least, to stabilize the neurological impairment. The complete resolution of neuroimaging involvement cannot be considered a therapeutic target, with some study^50^ reporting no significant changes in neuroimaging studies even when patients achieved a clinical response. In contrast, we have reported neuroimaging relapses in some asymptomatic patients who achieved a complete clinical response, suggesting that a close neurological follow-up should be carried out in patients with CNS neurosarcoidosis. The review by Fritz et al.^31^ showed a favourable outcome in 71% of cases after receiving first line therapies (overwhelmingly corticoids), while two additional series^24,27^ reported that at the last visit, only 16 (21%) were asymptomatic, with 36 (48%) having minor sequelae and 23 (31%) remaining with moderate/severe impairment.

Similarly to previous studies, which were also descriptive series of cases, our study has several potential limitations inherent to this uncontrolled design. As all retrospective analysis, it may suffer from potential selection biases. In addition, although we believe that the statistical approach that we used to compare the different neurological subsets is valid, we acknowledge that the sample sizes are small and multiple comparisons were made, and therefore, the statistically-significant findings should be interpreted with caution. In contrast, we think that a significant strength has been the use of the most recent internationally-proposed set of criteria that may have contributed to a more precise definition and classification of neurosarcoidosis in our cohort. Until now, the majority of criteria are derived from proposals from single institutions on the basis of a description of a personal series of cases^51–53^, expert opinions^21,54,55^ or scientific societies of single countries^56^. Therefore, there was a wide heterogeneity in the patients classified as neurosarcoidosis including in the series reported until now^10^, with some series even considering patients with muscular sarcoidosis^24,29^ or with ocular involvement/uveitis^23,27^ as neurosarcoidosis. Probably, the best classification approach was made by the WASOG consensus group^13^ who defined as highly/at least probable neurosarcoidosis the granulomatous inflammation of the meninges, brain, ventricular (CSF) system, cranial nerves, pituitary gland, spinal cord, cerebral vasculature or nerve roots, especially in the presence of abnormal MRI characteristic of neurosarcoidosis (abnormal enhancement following the administration of gadolinium) or a cerebrospinal fluid exam demonstrating inflammation; however, PNS involvement was excluded from the WASOG classification. Therefore, we believe that the use of the criteria recently proposed by Stern et al.^10^ has contributed significantly to obtain a more homogeneous picture of the involvement of the nervous system by sarcoidosis in one of the largest series of reported cases.

In a large cohort of patients from southern Europe, 5% fulfilled the criteria of neurosarcoidosis recently proposed by Stern et al.^10^. Among the two main subsets of neurological involvement, CNS involvement prevails significantly over PNS involvement, and both conditions do not overlap in any patient. The systemic phenotype associated to each involvement is clearly differentiated, with a lower thoracic and greater ocular and glandular expression in patients with CNS involvement, and a greater hepatosplenic expression in those with PNS involvement. This differentiated systemic expression can be helpful not only in the early identification of neurological involvement, but also in the systemic evaluation of patients diagnosed with neurosarcoidosis.

## Supplementary Information

Supplementary Information 1.

Supplementary Information 2.
