# Supplementary material for: Clinical characterization and outcomes of 85 patients with neurosarcoidosis
Source: Sci Rep. 2021 Jul 2;11:13735. doi: 10.1038/s41598-021-92967-6 (PMC8253777; doi:10.1038/s41598-021-92967-6)
Supplement: Supplementary file 1 — Supplementary Information 1. [file 41598_2021_92967_MOESM1_ESM.pptx]

## Slide 1
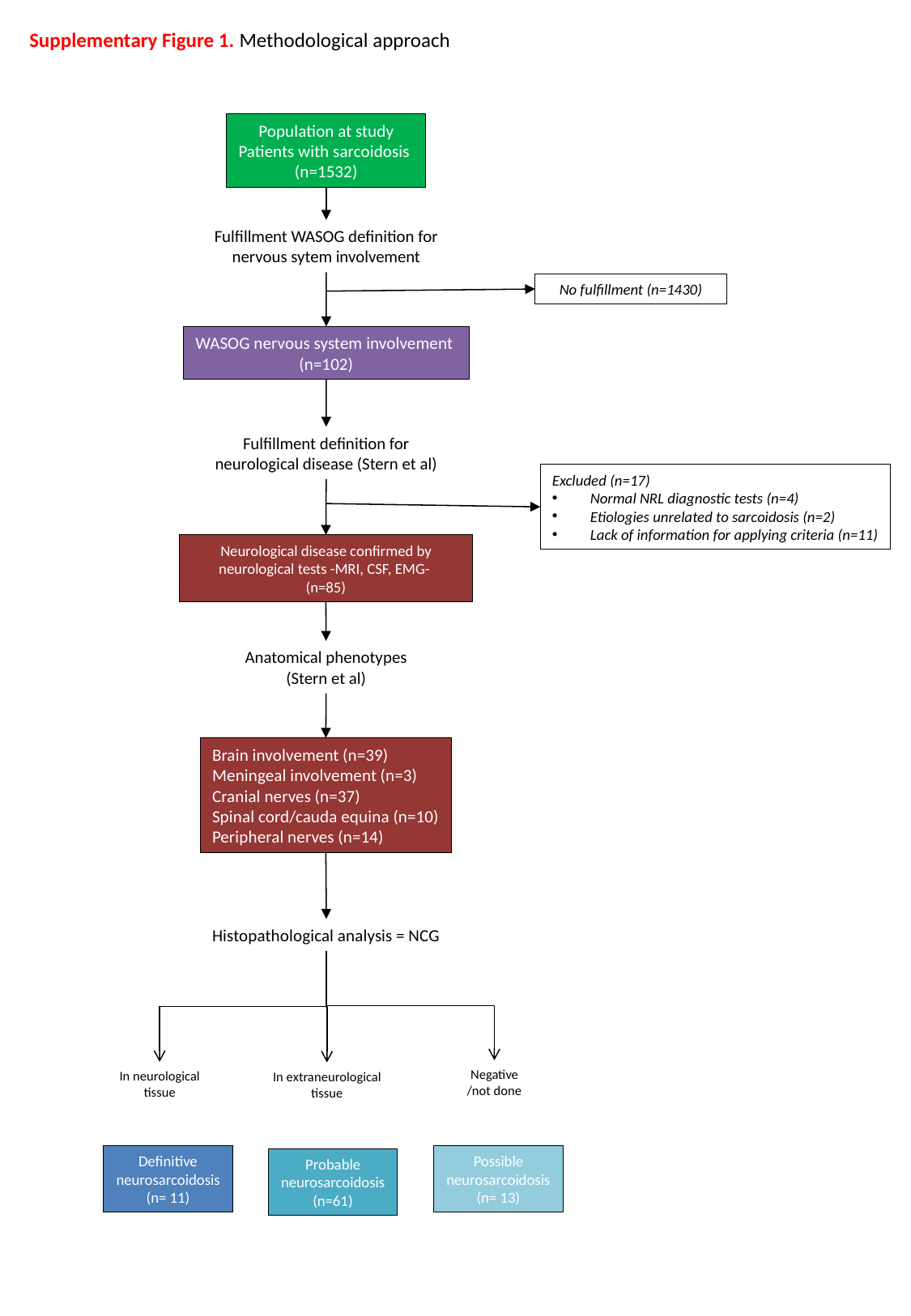

Supplementary Figure 1. Methodological approach
Population at study
Patients with sarcoidosis
(n=1532)
Fulfillment WASOG definition for nervous sytem involvement
No fulfillment (n=1430)
WASOG nervous system involvement
(n=102)
Fulfillment definition for neurological disease (Stern et al)
Excluded (n=17)
Normal NRL diagnostic tests (n=4)
Etiologies unrelated to sarcoidosis (n=2)
Lack of information for applying criteria (n=11)
Neurological disease confirmed by neurological tests -MRI, CSF, EMG-
(n=85)
Anatomical phenotypes (Stern et al)
Brain involvement (n=39)
Meningeal involvement (n=3)
Cranial nerves (n=37)
Spinal cord/cauda equina (n=10)
Peripheral nerves (n=14)
Histopathological analysis = NCG
Negative
/not done
In neurological tissue
In extraneurological tissue
Definitive neurosarcoidosis (n= 11)
Possible neurosarcoidosis (n= 13)
Probable neurosarcoidosis (n=61)
